# A causal relationship between particulate matter 2.5 and obesity and its related indicators: a Mendelian randomization study of European ancestry

**DOI:** 10.3389/fpubh.2024.1366838

**Published:** 2024-06-14

**Authors:** Tian qiang Wu, Xinyu Han, Chun yan Liu, Na Zhao, Jian Ma

**Affiliations:** ^1^Department of First Clinical Medical College, Heilongjiang University of Chinese Medicine, Harbin, China; ^2^Department of Endocrinology, The First Affiliated Hospital of Heilongjiang University of Chinese Medicine, Harbin, China

**Keywords:** Mendelian randomization, PM_2.5_, obesity, visceral adipose tissue, fibroblast growth factor 21, HbA1c, causality

## Abstract

**Background:**

In recent years, the prevalence of obesity has continued to increase as a global health concern. Numerous epidemiological studies have confirmed the long-term effects of exposure to ambient air pollutant particulate matter 2.5 (PM_2.5_) on obesity, but their relationship remains ambiguous.

**Methods:**

Utilizing large-scale publicly available genome-wide association studies (GWAS), we conducted univariate and multivariate Mendelian randomization (MR) analyses to assess the causal effect of PM_2.5_ exposure on obesity and its related indicators. The primary outcome given for both univariate MR (UVMR) and multivariate MR (MVMR) is the estimation utilizing the inverse variance weighted (IVW) method. The weighted median, MR-Egger, and maximum likelihood techniques were employed for UVMR, while the MVMR-Lasso method was applied for MVMR in the supplementary analyses. In addition, we conducted a series of thorough sensitivity studies to determine the accuracy of our MR findings.

**Results:**

The UVMR analysis demonstrated a significant association between PM_2.5_ exposure and an increased risk of obesity, as indicated by the IVW model (odds ratio [OR]: 6.427; 95% confidence interval [CI]: 1.881–21.968; *P*_FDR_ = 0.005). Additionally, PM_2.5_ concentrations were positively associated with fat distribution metrics, including visceral adipose tissue (VAT) (OR: 1.861; 95% CI: 1.244–2.776; *P*_FDR_ = 0.004), particularly pancreatic fat (OR: 3.499; 95% CI: 2.092–5.855; PFDR =1.28E-05), and abdominal subcutaneous adipose tissue (ASAT) volume (OR: 1.773; 95% CI: 1.106–2.841; *P*_FDR_ = 0.019). Furthermore, PM_2.5_ exposure correlated positively with markers of glucose and lipid metabolism, specifically triglycerides (TG) (OR: 19.959; 95% CI: 1.269–3.022; *P*_FDR_ = 0.004) and glycated hemoglobin (HbA1c) (OR: 2.462; 95% CI: 1.34–4.649; *P*_FDR_ = 0.007). Finally, a significant negative association was observed between PM_2.5_ concentrations and levels of the novel obesity-related biomarker fibroblast growth factor 21 (FGF-21) (OR: 0.148; 95% CI: 0.025–0.89; *P*_FDR_ = 0.037). After adjusting for confounding factors, including external smoke exposure, physical activity, educational attainment (EA), participation in sports clubs or gym leisure activities, and Townsend deprivation index at recruitment (TDI), the MVMR analysis revealed that PM_2.5_ levels maintained significant associations with pancreatic fat, HbA1c, and FGF-21.

**Conclusion:**

Our MR study demonstrates conclusively that higher PM_2.5_ concentrations are associated with an increased risk of obesity-related indicators such as pancreatic fat content, HbA1c, and FGF-21. The potential mechanisms require additional investigation.

## 1 Introduction

Obesity is a common chronic metabolic disease that is primarily characterized by excessive total fat content or local increase and abnormal distribution. It can be complicated by type 2 diabetes, hyperlipidemia, nonalcoholic fatty liver, depression, osteoarthritis, asthma, and other multisystem diseases, which have severe effects on quality of life and life expectancy (1). Since 1975, the number of obese patients in the globe has nearly doubled, according to a report published by the Globe Health Organization in 2021. In 2016, more than 39 percent of adults aged 18 and older were overweight, 13 percent of them were obese, and the number of obese children and adolescents reached an alarming 124 million ^1^(2), rendering obesity a severe global public health problem. For the diagnosis of obesity, body mass index (BMI) is the critical marker, and fat distribution such as visceral adipose tissue (VAT), abdominal subcutaneous adipose tissue (ASAT), pancreas fat, glycolipid metabolism indicators like glycated hemoglobin (HbA1c), triglycerides (TG), and biomarker fibroblast growth factor 21 (FGF-21) is vital in the early screening and evaluation of obesity and obesity-related diseases. VAT is the visceral adipose tissue surrounding the abdominal organs, which is the body’s primary site for energy storage. Its excessive accumulation is linked to a number of metabolic disorders, including insulin resistance and inflammatory response (3).

ASAT is the main subcutaneous adipose reservoir. Although it has fewer adverse effects on metabolic health than VAT, it has a stronger connection with the physical characteristics of obesity (4). Pancreas fat refers to the fat content of pancreatic tissue, and its abnormal aggregation can disrupt the function of the islets of Langerhans β Cells, resulting in a disorder of blood glucose regulation (5). HbA1c is a product of the combination of hemoglobin and glucose, which can reflect the average blood sugar level over the past 2–3 months. In obese patients, an increase in HbA1c may indicate a decrease in insulin sensitivity and impaired glucose metabolism (6). TG is the predominant form of lipid in plasma, which is generally synthesized in the liver and stored in adipocytes. TG abnormalities are frequently associated with obesity and metabolic syndrome (7). FGF defines a family of proteins essential for cell proliferation, differentiation, migration, and survival. It is strongly connected to the proliferation and differentiation of adipocytes, and its abnormal expression can contribute to the development of obesity and its related complications (8). The etiology and pathogenesis of obesity are still unclear, and current research has attributed it to dietary habits, behavior, genetics, and socioeconomic and environmental factors (9–11).

With the acceleration of urbanization on a global scale, emissions of pollutants from industrial production and transportation continue to rise, and the impact of air pollution on obesity is becoming increasingly significant. Particulate matter, the primary component of air pollution, alludes to the suspended and dispersed solid or liquid particles. According to the aerodynamic diameter, it can be divided into PM_2.5_, PM_10_, and PM_0.1_ (12). PM_2.5_ is defined as particles in the atmosphere with a diameter of _2.5_ microns or less, which have a small particle size, a large surface area, and a high level of activity. They are prone to transporting toxic substances and can linger in the atmosphere for a long time. After inhalation, they can further deposit in the alveoli, as well as penetrate the capillaries and systemic circulation (13). Thus, PM_2.5_ has a more significant negative impact on environmental quality and human health.

In recent years, mounting evidence has demonstrated that PM_2.5_ is a significant contributor to overweight, obesity, and endocrine and metabolic disorders tied to obesity. A large longitudinal cohort study involving over 3.9 million US veterans over an 8-year follow-up period revealed a 10-g/m3 higher average annual PM_2.5_ concentration was positively correlated with clinical risk of obesity (hazard ratio (HR) = 1.08, 95% confidence interval (CI): 1.06–1.11) and the risk of a 10-pounds (4.54 kg) weight gain (HR = 1.07, 95% CI: 1.06–1.08) (14). Another large-scale cross-sectional study involving 47,204 adults in 13 provinces of China (15) indicated that every 10 μg/m^3^ increase in PM_2.5_ is related to a higher incidence of obesity (OR = 1.12, 95% CI: 1.09–1.14) as well as abdominal adiposity (OR = 1.10, 95% CI =1.07–1.13). In addition, longitudinal cohort research from Taiwan (16) demonstrated that an increase of 10 μg/m^3^ in the annual average concentration of PM_2.5_ is linked to an increase in TG (adjusted hazard ratio (aHR) =1.17, 95% CI: 1.11–1.23) and a rise in fasting blood glucose (aHR = 1.15, 95% CI: 1.10–1.20) suggesting a relationship between PM_2.5_ and impaired glucose and lipid metabolism. A few cross-sectional studies, however, have shown that exposure to environmental pollutants such as PM_2.5_ does not substantially contribute to obesity and related lipid metabolism indicators (17, 18). The inconsistency in the results of observational research can be due to sample size bias and residual confounding factors. At present, the causality between PM_2.5_ and obesity and its related indicators is still ambiguous, and further proof is required for confirmation.

MR is an epidemiological technique designed to overcome the limitations of observational investigations, and it has been extensively utilized in a variety of studies. The central concept of MR is to infer the causal relationship between exposure and outcome employing genetic variation as Instrumental variables (IVs). Given the fact that genetic variation is assigned randomly by parents to offspring at conception and is relatively independent of factors such as social environment and personal lifestyle, it is possible to avoid the influence of remaining confounding factors or reverse causal relationships in observational studies and obtain more reliable findings (19, 20). MVMR is an emerging technology that can integrate the genetic variation of multiple risk factors into a single model and simultaneously evaluate the corresponding exposure in order to minimize the impact of mixed variables (21). This study implemented univariate and multivariate MR analyses to investigate the causal association between PM_2.5_ and obesity and the accompanying indicators.

## 2 Materials and methods

### 2.1 Study design

**Figure 1:**
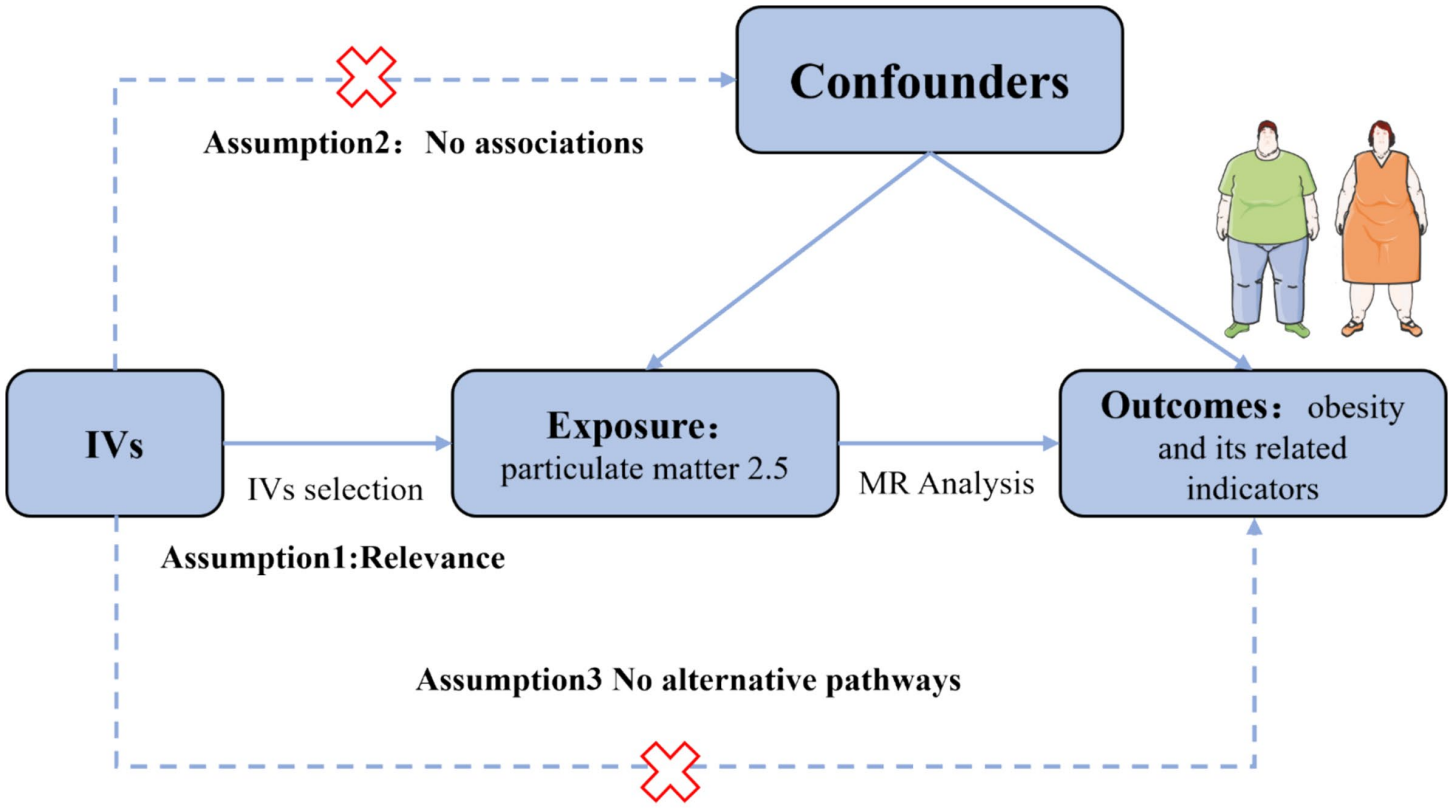
Assumption of the Mendelian randomization (MR) analysis for PM_2.5_ and obesity and its related indicators.

Utilizing publicly available data, we conducted a two-sample MR analysis to assess the causal relationship between PM_2.5_ and obesity and its related indicators. The methodology of this MR investigation is illustrated in Figure 1. The IVs chosen for causal estimation must satisfy three fundamental assumptions (22): Assumption 1, The IVs should be strongly related to PM_2.5_ (*p* < 5 × 10^−8^); Assumption 2: The correlation between IVs of PM_2.5_ and obesity or related indicators is unaffected by the presence of confounding factors; Assumption 3: The IVs only influences the risk of obesity and related indicators via PM_2.5_, rather than other channels. Prior observational clinical trials and MR studies have demonstrated that exposure to tobacco smoke, physical activity, EA, participation in sports clubs or gym leisure activities, and TDI are risk factors for the onset of obesity and impaired glucose and lipid metabolism (23–28). Consequently, we adjusted the genetic susceptibility of these five variables further using MVMR. Since our data is derived from publicly accessible GWAS aggregated statistical data, no ethical approval is required.

### 2.2 Data sources

The summary statistical data for PM_2.5_ originates from the MRC-IEU alliance (dataset ID: ieu-b-4879), which includes 423,796 participants of European ancestry. The Land Use Regression (LUR) model was utilized to assess the PM_2.5_ concentration near the participant’s residences (29). Obesity and its associated indicators (including VAT, ASAT, Pancreatic fat, Hba1c, TG, and FGF21) were chosen as outcomes. The GWAS summary data for obesity (8,908 cases and 209,827 controls) are available from the FinnGen consortium. This large public-private collaboration research project combines estimated genotype data derived from newly collected and legacy samples from the Finnish Biobank with digital record data from the Finnish Health Registry (30). The combined level data for VAT, ASAT, and pancreas fat were obtained from the study on deep learning algorithms conducted by Liu Y. et al. in 2021, which utilized abdominal magnetic resonance imaging (MRI) to determine the genetic structure of body composition (31). The dataset pertaining to HbA1c and TG originated from a GWAS study undertaken by Howe LJ et al., involving 182,416 and 69,360 European males and females, respectively. The IVs with regard to FGF21 were extracted from a GWAS study conducted by Gilly et al. (32), which identified 18,160,173 single nucleotide polymorphisms (SNPs) among 1,298 samples. In addition, aggregate data regarding external smoke exposure, physical activity, EA, participation in sports clubs or gym leisure activities, and TDI were obtained from the Neale Lab or MRC-IEU consortium. All participants in this study are of European descent. Table 1 provides a summary of all datasets included in this investigation.

**Table 1:** Details of studies included in Mendelian randomization (MR) analyses.

Traits	Author	GWAS ID	Sample size(cases/controls)	Number of SNPs	Sex	Ancestry	Year	PMID
Exposure								
PM2.5	Ben Elsworth	ukb-b-10817	423,796	9,851,867	Males and females	European	2018	27089921
Exposure to tobacco smoke outside home	Neale	ukb-a-20	286,550	10,894,596	Males and females	European	2017	NA
physical activity	Neale	ukb-a-485	335,599	10,894,596	Males and females	European	2017	NA
Year ended full time education	Ben Elsworth	ukb-b-2709	112,569	9,851,867	Males and females	European	2018	NA
Leisure/social activities: Sports club or gym	Ben Elsworth	ukb-b-4000	461,369	9,851,867	Males and females	European	2018	NA
TDI	Neale	ukb-a-44	336,798	10,894,596	Males and females	European	2017	NA
Outcomes								
Obesity	NA	finn-b-E4_OBESITY	8,908/209,827	16,380,465	Males and females	European	2021	NA
VAT	Liu Y	ebi-a-GCST90016671	32,860	9,275,407	NA	European	2021	34128465
ASAT	Liu Y	ebi-a-GCST90016672	32,860	9,275,407	NA	European	2021	34128465
Pancreas fat	Liu Y	ebi-a-GCST90016675	25,617	9,275,407	NA	European	2021	34128465
Triglycerides	Howe LJ	ieu-b-4850	78,700	7,892,037	Males and females	European	2022	NA
HbA1c	Howe LJ	ieu-b-4841	17,724	NA	Males and females	European	2022	NA
FGF21	Gilly A	ebi-a-GCST90010123	1,298	18,160,173	NA	European	2020	33303764

PM_2.5_, particulate matter 2.5; TDI, Townsend deprivation index at recruitment; VAT, visceral adipose tissue volume; ASAT, abdominal subcutaneous adipose tissue volume; HbA1c, glycosylated hemoglobin; FGF21, fibroblast growth factor 21 levels.

### 2.3 Selection and evaluation of instrumental variable

We implemented a specific procedure for selecting the IVs to fulfill the three critical assumptions of MR analysis. Firstly, genetic variations must be strongly associated with the exposure to satisfy the first hypothesis. We extracted SNPs strongly related to PM_2.5_ at the significance level of *p* < 5 × 10^−8^. To fulfill the requirements of hypothesis 2 in the MR framework, we established a stringent criterion (*r*^2^ < 0.001 and a clumping distance of 10,000 kb), ensuring that the selected IVs were conditionally independent. Only SNPs with the lowest *p*-values were retained to mitigate the impact of linkage disequilibrium (LD) among the SNPs (33). Furthermore, the potential pleiotropic effects were controlled by extracting the secondary phenotype of each SNP from PhenoScan V2 (34).^2^ It is widely recognized that parameters such as body mass index, waist circumference, and waist-to-hip ratio are strongly correlated with obesity, fat distribution, and glucose and lipid metabolism (35–38). Consequently, we eliminated any independent variables associated with these parameters or directly linked to outcome measures. This approach helps ensure that the IVs used in our MR study are specific to the exposure of interest and not influenced by other pathways that could bias the results. Ultimately, we extracted exposure IVs from the outcome data and conducted data harmonization to exclude SNPs with inconsistent exposure and outcome data alleles.

Variance (R2) and the F-statistic were utilized to evaluate the robustness of IVs in order to prevent bias from a weak tool. The F-statistic for each SNP is determined by the following formula: F = R^2^/(1-R^2^) [(N-K-1)/K], where N is the sample size, K is the total number of SNPs chosen for MR analysis, and R^2^ is the overall proportion of phenotypic variations explained by all SNPs in our MR model (39). R^2^ was estimated for all SNP through the given formula: R^2^ = Σ [2 × (1 – MAF) × MAF × β^2^/ (SE^2^ × N)]. MAF is the minor allele frequency for each SNP, and SE and β are the standard error and effect size coefficient, respectively. An F-statistic greater than 10 was regarded as adequate for the relationship between IVs and exposure to avoid weak tool bias from influencing the results of MR analyses (22).

### 2.4 Statistical analysis

The IVW method was utilized as the primary analysis. In addition, weighted median, MR Egger, and maximum likelihood were used to assess robustness effects. The IVW method is an expansion of the Wald ratio estimator based on meta-analytic principles, which can offer precise estimates in the absence of horizontal or balanced pleiotropy (40). With at least 50 percent of the weight of the analysis originating from valid IVs, the weighted median method is able to draw a trustworthy conclusion (41). Despite its low statistical power, the MR Egger method can detect potential pleiotropy and present estimates after controlling for multiple effects (42). The maximum likelihood method is analogous to the IVW method, where the results are unbiased, and the standard error is less than that of the IVW method under the assumption of no heterogeneity and horizontal pleiotropy (43). On the basis of prior research (23–28), we adjusted for external smoke exposure, physical activity, EA, participation in sports clubs or gym leisure activities, and TDI in MVMR to illustrate a causal relationship between PM_2.5_ and obesity and its related indicators. IVW and MR-Lasso (44) were included in the approaches we employed to execute MVMR.

In this investigation, numerous sensitivity analyses were performed to assure the stability and dependability of the MR results. First, Cochran’s Q test was applied to evaluate the heterogeneity between SNPs, where a *p*-value greater than 0.05 indicated no heterogeneity. Secondly, the MR-Egger intercept was utilized to quantify the horizontal pleiotropy of IVs. Thirdly, we performed the leave-one-out analysis to check whether any single SNP drove the MR results. Finally, we conducted the MR-PRESSO to detect potential outlier SNPs (45). A two-sided *p*-value less than 0.05 was regarded as statistically significant. In order to account for multiple hypothesis testing, we calculated the adjusted *p* values (*q* values) for the false discovery rate (FDR) in the significant IVW MR analyses. We used the sequential *p*-value approach suggested by Benjamini and Hochberg (46). A *q* value less than or equal to 5% was deemed significant. The R packages TwoSampleMR (version 0.5.6) and MVMR (version 0.3) served as tools to undertake MR analyses. All data analyses were conducted utilizing version 4.3.1 of R.

## 3 Result

### 3.1 Genetic instruments

In the present research, 6, 6, 6, 6, 4, 6, and 7 SNPs were ultimately identified as the IVs for PM_2.5_ to assess the associations between PM_2.5_ and Obesity, VAT, ASAT, pancreas fat, TG, HbA1c and FGF21, respectively (Supplementary Tables S1–S7). The F statistic for each of these genetic variants was greater than 10, indicating a low probability of mild instrumental bias.

### 3.2 Estimated causal effect of PM_2.5_ on obesity and its related indicators

**Figure 2:**
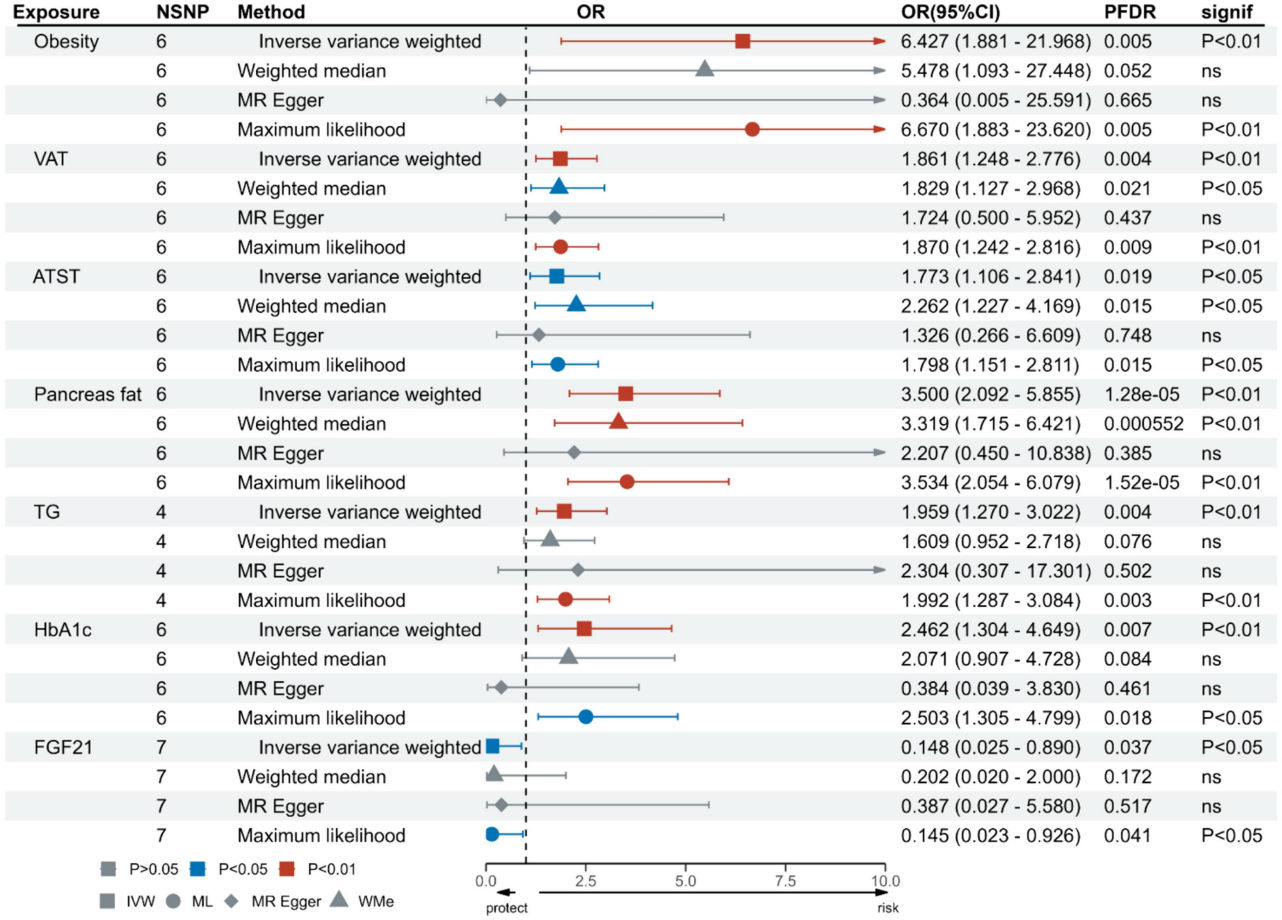
Association of genetically predicted PM_2.5_ and obesity and its related indicators.

**Figure 3:**
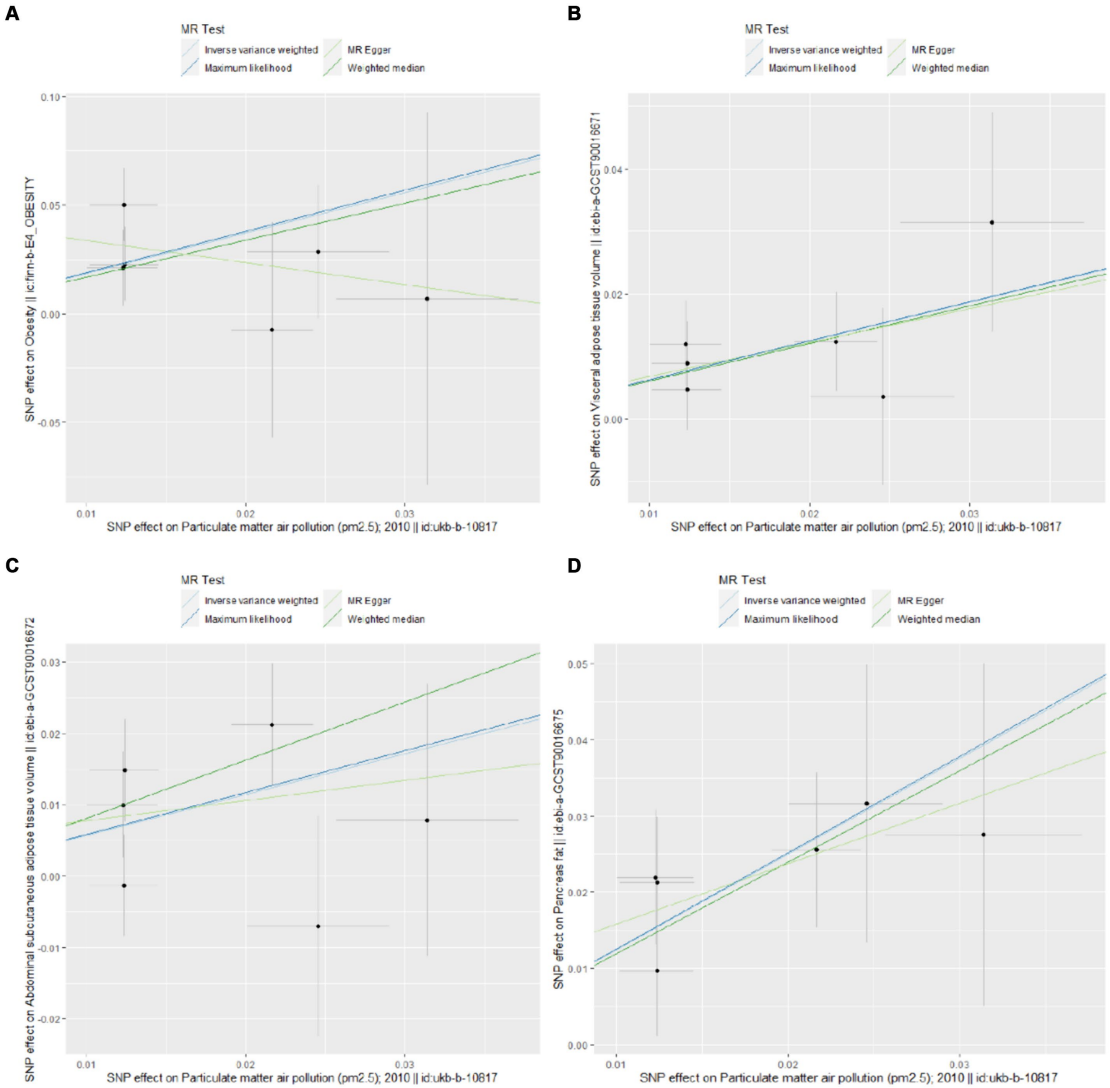
Scatter plots for Mendelian randomization (MR) analyses of the correlation between PM_2.5_ and obesity and its related indicators. **(A)** Obesity; **(B)** VAT; **(C)** ASAT; **(D)** pancreatic fat.

**Figure 4:**
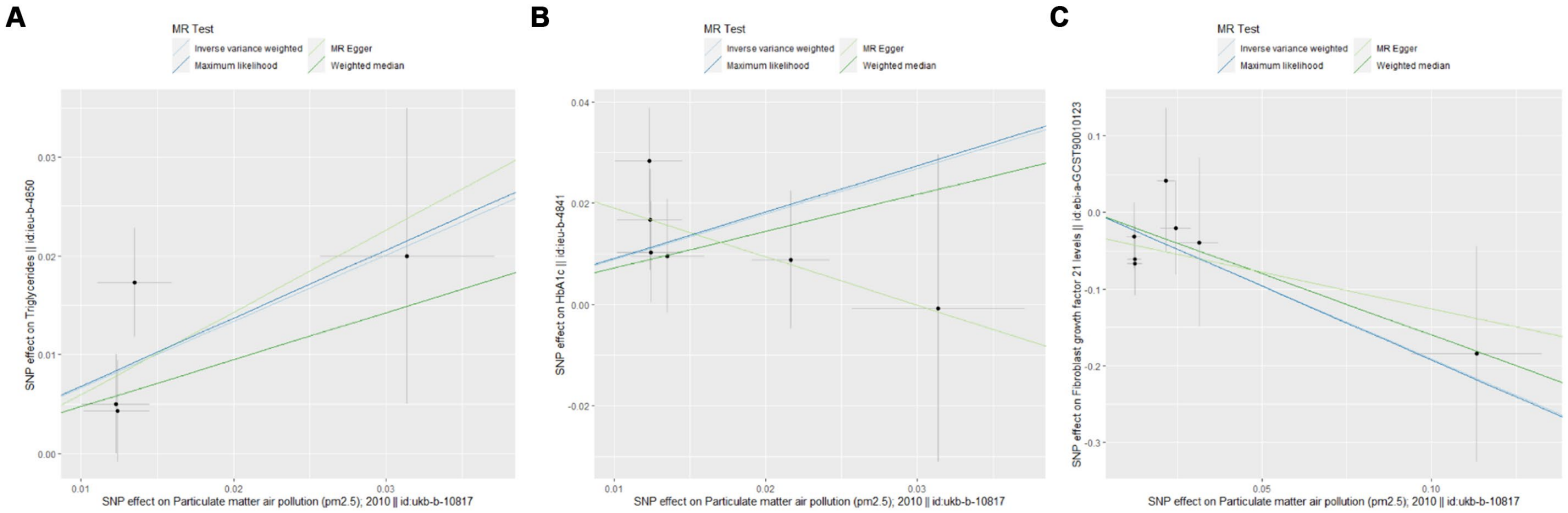
Scatter plots for Mendelian randomization (MR) analyses of the correlation between PM_2.5_ and obesity and its related indicators. **(A)** TG; **(B)** HbA1c; **(C)** FGF21.

The UVMR analysis demonstrated a significant association between PM_2.5_ exposure and an increased risk of obesity, as indicated by the IVW model (OR: 6.427; 95% CI: 1.881–21.968; *P*_FDR_ = 0.005). Additionally, PM_2.5_ concentrations were positively associated with fat distribution metrics, including VAT (OR: 1.861; 95% CI: 1.244–2.776; *P*_FDR_ = 0.004), particularly pancreatic fat (OR: 3.499; 95% CI: 2.092–5.855; PFDR =1.28E-05), and ASAT volume (OR: 1.773; 95% CI: 1.106–2.841; *P*_FDR_ = 0.019). Furthermore, PM_2.5_ exposure correlated positively with markers of glucose and lipid metabolism, specifically TG (OR: 19.959; 95% CI: 1.269–3.022; *P*_FDR_ = 0.004) and HbA1c (OR: 2.462; 95% CI: 1.34–4.649; *P*_FDR_ = 0.007). Finally, a significant negative association was observed between PM_2.5_ concentrations and levels of the novel obesity-related biomarker FGF-21 (OR: 0.148; 95% CI: 0.025–0.89; *P*_FDR_ = 0.037). Figure 2 depicts the causal associations between genetically predicted PM_2.5_ and the risk of obesity and its related indicators. The scatter plots of the association between PM_2.5_ and obesity and its related indicators are shown in Figures 3, 4.

In the MVMR-IVW analysis adjusting for external smoke exposure, physical activity, EA, participation in sports clubs or gym leisure activities, and TDI, the causal relationship between PM_2.5_ and pancreas fat (OR: 3.612; 95%CI: 1.893–6.892; *p* = 9.82E-05), HbA1c (OR: 5.429; 95%CI: 2.327–12.665; *p* = 9.00E-05) or FGF21 (OR: 0.162; 95%CI: 0.034–0.776; *p* = 0.023) remained significant. The MVMR-Lasso technique results additionally revealed that a causal link between PM_2.5_ and pancreas fat or HbA1c still exists (Supplementary Table S8). The association between PM_2.5_ and Obesity, VAT, ASAT, or TG did not persist, however, after adjusting for the five confounding factors mentioned above. Table 2 presents the MVMR-IVW results in detail.

**Table 2:** Causal estimates of PM_2.5_ on obesity and its related indicators in MVMR-IVW.

Outcome	MVMR	Method	*p* value	OR	Low	UP
Obesity	IVW	Adjusted for Exposure to tobacco smoke	0.598	1.254	0.541	2.907
		Adjusted for Strenuous sports in last 4 weeks	0.498	1.303	0.606	2.800
		Adjusted for Year ended full time education	0.454	1.368	0.602	3.106
		Adjusted for Sports club or gym activities	0.606	1.231	0.559	2.710
		Adjusted for Townsend deprivation index	0.886	0.931	0.349	2.481
		Adjusted for all	0.401	1.642	0.516	5.228
VAT	IVW	Adjusted for Exposure to tobacco smoke	2.44E-06*	1.799	1.409	2.297
		Adjusted for Strenuous sports in last 4 weeks	0.011*	1.737	1.137	2.651
		Adjusted for Year ended full time education	4.03E-12*	2.028	1.661	2.476
		Adjusted for Sports club or gym activities	0.018*	1.621	1.085	2.421
		Adjusted for Townsend deprivation index	0.326	1.263	0.793	2.012
		Adjusted for all	0.233	1.373	0.816	2.311
ATST	IVW	Adjusted for Exposure to tobacco smoke	0.203	1.408	0.832	2.382
		Adjusted for Strenuous sports in last 4 weeks	0.454	1.289	0.664	2.501
		Adjusted for Year ended full time education	0.001*	1.840	1.268	2.670
		Adjusted for Sports club or gym activities	0.059	1.555	0.984	2.458
		Adjusted for Townsend deprivation index	0.887	0.963	0.571	1.622
		Adjusted for all	0.700	1.130	0.608	2.101
Pancreas fat	IVW	Adjusted for Exposure to tobacco smoke	5.42E-09*	3.116	2.127	4.565
		Adjusted for Strenuous sports in last 4 weeks	3.40E-10*	3.382	2.312	4.947
		Adjusted for Year ended full time education	5.43E-18*	3.347	2.545	4.402
		Adjusted for Sports club or gym activities	2.88E-06*	3.029	1.904	4.817
		Adjusted for Townsend deprivation index	7.10E-04*	3.388	1.672	6.868
		Adjusted for all	9.82E-05*	3.612	1.893	6.892
TG	IVW	Adjusted for Exposure to tobacco smoke	0.038*	1.665	1.030	2.691
		Adjusted for Strenuous sports in last 4 weeks	0.010*	1.720	1.136	2.640
		Adjusted for Year ended full time education	0.878	1.047	0.580	1.891
		Adjusted for Sports club or gym activities	3.13E-04*	2.775	1.593	4.835
		Adjusted for Townsend deprivation index	0.180	1.541	0.819	2.898
		Adjusted for all	0.597	1.306	0.486	3.514
HbA1c	IVW	Adjusted for Exposure to tobacco smoke	0.002*	3.095	1.494	6.411
		Adjusted for Strenuous sports in last 4 weeks	0.003*	3.154	1.464	6.794
		Adjusted for Year ended full time education	2.00E-04*	3.058	1.686	5.545
		Adjusted for Sports club or gym activities	3.00E-04*	2.775	1.593	4.835
		Adjusted for Townsend deprivation index	1.90E-04*	5.240	2.197	12.497
		Adjusted for all	9.00E-05*	5.429	2.327	12.665
FGF21	IVW	Adjusted for Exposure to tobacco smoke	0.003*	0.108	0.025	0.469
		Adjusted for Strenuous sports in last 4 weeks	0.010*	0.138	0.030	0.628
		Adjusted for Year ended full time education	0.053	0.230	0.052	1.018
		Adjusted for Sports club or gym activities	0.001*	0.128	0.036	0.453
		Adjusted for Townsend deprivation index	0.023*	0.162	0.034	0.776

PM_2.5_, particulate matter 2.5; VAT, visceral adipose tissue volume; ASAT, abdominal subcutaneous adipose tissue volume; HbA1c, glycosylated hemoglobin; FGF21, fibroblast growth factor 21 levels; MVMR, Multivariable Mendelian randomization; SE, standard error; OR, odds ratio; CI, confidence interval; *indicates that the relationship has statistical significance.

The Cochran’s Q test revealed no evidence of heterogeneity, and the MR-Egger intercept test discovered no indication of horizontal pleiotropy in the MR analysis results. In addition, MR-PRESSO failed to detect any SNPs that were outliers. The results of the sensitivity analysis are presented in Table 3. The leave-one-out plots offer additional evidence for the robustness of our results and imply that the effects of a single SNP were unlikely to influence causal estimates (Supplementary Figures S1–S7).

**Table 3:** Heterogeneity, horizontal pleiotropy, and MR-PRESSO tests of the associations between PM_2.5_ and obesity and its related indicators.

Outcomes	Pleiotropy test			Heterogeneity test						MR-PRESSO
	MR-Egger			MR-Egger			Inverse-variance weighted			Global test
	Intercept	SE	*p*	*Q*-value	Q-df	*Q*-pval	*Q*-value	Q-df	*Q*-pval	*p* value
Obesity	0.044	0.032	0.239	2.294	4	0.682	4.204	5	0.520	0.563
VAT	0.001	0.010	0.905	1.799	4	0.773	1.816	5	0.874	0.899
ASAT	0.005	0.013	0.727	5.689	4	0.224	5.888	5	0.317	0.349
Pancreas fat	0.007	0.013	0.581	1.423	4	0.840	1.783	5	0.878	0.908
Triglycerides	−0.002	0.014	0.885	3.236	2	0.198	3.280	3	0.350	0.428
HbA1c	0.018	0.011	0.198	1.913	4	0.752	4.627	5	0.463	0.483
FGF21	−0.030	0.032	0.385	2.037	5	0.844	2.942	6	0.816	0.870

PM_2.5_, particulate matter 2.5; VAT, visceral adipose tissue volume; ASAT, abdominal subcutaneous adipose tissue volume; HbA1c, glycosylated hemoglobin; FGF21, fibroblast growth factor 21 levels; MR-PRESSO, Mendelian randomization pleiotropy residual sum and outlier; Q-value, the statistics of Cochran’s Q test; SE, standard error.

## 4 Discussion

This study utilized MR technology to evaluate the causal relationship between the air pollutant PM_2.5_ and obesity, along with related indicators. After applying the FDR correction, the UVMR results indicate a significant causal relationship between exposure to PM_2.5_ and an increased susceptibility to obesity in the European population. Furthermore, supplementary MR analysis revealed a positive correlation between PM_2.5_ concentration and body fat distribution, including VAT, particularly pancreatic adipose tissue, and ASAT volume. Moreover, there is a direct link between exposure to PM_2.5_ and increased levels of TG and HbA1c. Conversely, a significant negative link was observed between PM_2.5_ concentration and the level of the novel obesity-related biomarker FGF-21. After adjusting for confounding factors such as external smoke exposure, physical activity, EA, participation in sports clubs or gym leisure activities, and TDI, MVMR analysis showed that PM_2.5_ levels maintained a significant association with pancreatic fat, HbA1c, and FGF-21.

Our UVMR research results offer genetic evidence for the causal relationship between PM_2.5_ exposure and the likelihood of obesity. In additional MR analyses of PM_2.5_ and fat distribution, we identified a positive correlation between PM_2.5_ concentrations and VAT, ASAT, or pancreatic fat. These findings are compatible with previous observational epidemiological studies.

A cross-sectional study conducted in Spain examined the relationship between PM_2.5_ levels and the prevalence of overweight in young people aged 2–14. It found that compared to areas with low PM_2.5_ levels, areas with moderate PM_2.5_ levels had a 23% higher risk of overweight, while areas with high PM_2.5_ levels had a 35% higher risk (47). An analysis of data from a study involving 11,766 participants found an intense connection between exposure to PM_2.5_ and visceral fat index (VFI) in middle-aged and older adult individuals. The highest quartile OR was 1.10 (95% CI, 1.07, 1.13) (48). A separate cohort study, consisting of 38,824 participants aged 18–79, also arrived at comparable conclusions. It observed that the VFI exhibited a rising pattern as the amount of PM_2.5_ increased (49).

An investigation carried out in Chongqing, China, discovered a distinct correlation between exposure to PM_2.5_ and childhood obesity, specifically centripetal obesity, as assessed by the waist-to-height ratio (WHtR). This study validates that environmental pollution has a cumulative effect on obesity, specifically the buildup of abdominal fat, in young persons from China (50). Additionally, a comprehensive nationwide longitudinal study conducted in China also showed a clear link between air pollution and the heightened susceptibility of older adult individuals to both general obesity and abdominal obesity. With each standard deviation increase in the Average Air Quality Index (AQI), the likelihood of becoming centripetal obesity increases by 2.8% (95%CI, 1.7, 3.9%), and the probability of developing abdominal obesity increases by 6.2% (95%CI, 4.4, 8.0%) (51).

Currently, the majority of studies focus on investigating the influence of PM_2.5_ on the overall amount of visceral fat. However, there is insufficient study on the correlation between PM_2.5_ levels and specific types of visceral fat, such as pancreatic fat. The excessive accumulation of lipids in the pancreas is accompanied by a decline in the cellular activity of pancreatic islets (52). Prior research has demonstrated a negative correlation (*p* < 0.03) between the average amount of fat in the pancreas, as measured by magnetic resonance imaging (MRI), and markers of insulin secretion based on oral glucose tolerance tests (OGTT). The results of the subsequent stepwise multiple regression analysis indicate that pancreatic fat is more closely linked with reduced insulin secretion function compared to other visceral lipids, such as liver fat (53). Animal studies have demonstrated that exposure to PM_2.5_ can reduce the expression of glucose transporter 2 (GLUT 2) in pancreatic tissue of rats with gestational diabetes (GDM), thereby increasing the likelihood of pancreatic lipid deposition, tissue damage and elevated blood sugar levels (49). The research above indirectly demonstrates the correlation between PM_2.5_ levels and the abnormal accumulation of fat in the pancreas. A cohort research (54) published in 2017 indicated that exposure to high amounts of PM_2.5_ had a deleterious impact on insulin sensitivity (SI) and β Cellular function in children and is not tied to obesity markers such as body fat percentage. In the MVMR model, after adjusting for external smoke exposure, physical exercise, education level, gym leisure activities, and TDI, a significant relationship between PM_2.5_ concentration and pancreatic fat remains. However, the relationships between PM_2.5_ concentration and obesity, VAT, and ASAT are no longer significant. The TDI, which reflects socio-economic status, is the main confounding factor, closely related to both PM_2.5_ exposure and obesity (55, 56). The discrepancies between MR analysis results and observational research can be attributed to the influence of confounding factors. Therefore, further research is needed to explore the relationship between PM_2.5_ concentration and obesity, VAT, and ASAT.

Previous studies have drawn contradictory conclusions about the relationship between PM_2.5_ and TG levels. A recent cohort study has shown a correlation between air pollution, including PM_2.5,_ and a higher likelihood of blood lipid abnormalities (OR = 1.14, 95% CI: 1.10, 1.18). It found that for every 10% increase in PM_2.5_ concentration (measured in μg/m3), there was a corresponding 3.04% rise in TG levels (95% CI: 2.61, 3.47%) (57). Nevertheless, specific research has indicated that prolonged exposure to air pollution, specifically PM_2.5_, is solely linked to raised levels of total cholesterol (TC) and a higher incidence of hypercholesterolemia in children and adolescents. However, there is no clear association between air pollution and other lipid abnormalities, such as TG and low-density lipoprotein cholesterol (LDL-C) (58). This aligns with the findings of our research. In our UVMR results, there is a direct correlation between PM_2.5_ and elevated TG levels. However, this relationship becomes insignificant when considering external smoke exposure, physical exercise, education level, gym leisure, and TDI in the MVMR model. The difference between MR analysis and observational data may be attributed to confounding variables. Therefore, it is necessary further to investigate the relationship between PM_2.5_ concentration and TG.

Our investigation on MVMR revealed a significant correlation between PM_2.5_ levels and HbA1c after taking into consideration other influencing factors. A recent statewide cohort research conducted in China has indicated a positive correlation between increased exposure to PM_2.5_ and higher levels of HbA1c. In the primary model, a 10 μg/m3 increase in PM_2.5_ exposure concentration corresponded with a 0.016 mmol/L increase in HbA1c levels (59). Another study conducted in South Korea, a developed country, also found that an increase in PM_2.5_ levels by one quartile range (IQR) coincided with a 0.34% increase in HbA1c levels (95% CI: 0.04, 0.63) (60). Added to that, we discovered an inverse correlation that is nominally significant between the content of PM_2.5_ and FGF-21 in both the UVMR and MVMR models. FGF-21, belonging to the fibroblast growth factor family, exerts regulatory metabolic effects, including cholesterol reduction, blood sugar reduction, insulin resistance improvement, and weight reduction. It is frequently linked with the presence of chronic metabolic disorders related to obesity (61). Although empirical research exploring the correlation between PM_2.5_ levels and FGF-21 is currently lacking, our MR results suggest that improving air quality and reducing PM_2.5_ concentrations could have beneficial effects on mitigating obesity-related chronic metabolic disorders.

At present, the exact mechanism of the association between PM_2.5_ exposure and obesity or glucose and lipid metabolism disorders is not clear. However, existing research suggests that this correlation may be related to the following four potential mechanisms.

Firstly, PM_2.5_ can trigger systemic and local chronic inflammatory responses, which are vital factors that contribute to metabolic disorders and obesity (62, 63). An animal study has demonstrated that PM_2.5_ exposure can induce glucose and lipid metabolism disorders in both normal healthy and diabetic model mice. This metabolic damage is consistent with an increase in inflammatory responses in the respiratory system, circulatory system, and VAT, characterized by the release of interleukin (IL)-6 and tumor necrosis factor-alpha (TNF-α) in the lungs, serum, and VAT. Furthermore, the use of AMPK activators to inhibit the release of inflammatory cytokines has been shown to alleviate PM_2.5_-induced metabolic disorders (64). Another animal study confirmed that the activation of NLRP3 inflammasomes and the increase in levels of related inflammatory cytokines, including IL-18 and IL-1β, are the main mechanisms behind the aggravation of PM_2.5_-related metabolic disorders in diabetic model mice (65).

Secondly, the oxidative stress (OS) response plays an essential mediating role in metabolic disorders caused by PM_2.5_ exposure (66). PM_2.5_ contains various pro-oxidant molecules, and its toxicity is linked to its ability to produce reactive oxygen species (ROS). Exposure to PM_2.5_ can induce oxidative stress in different tissues and cell lines (67). Animal research data suggests that exposure to PM_2.5_ can reduce vascular insulin sensitivity by inducing oxidative stress in the lungs, leading to insulin resistance. Antioxidant treatment or overexpression of lung-specific extracellular superoxide dismutase (ecSOD) can alleviate insulin resistance in PM_2.5_-exposed mice (68). On top of that, a recently published study revealed that PM_2.5_ greatly enhances xanthine levels in both brown adipose tissue (BAT) and white adipose tissue (WAT) in male db/db mice, provoking OS in the adipose tissue (69). Higher levels of ROS can promote the accumulation of TG in WAT and hinder its breakdown, bringing about an increase in WAT content and a decrease in BAT content, consequently impacting energy metabolism, diminishing insulin sensitivity, and worsening blood glucose disorders (69). PM_2.5_ exposure can harm skeletal muscle function by impairing mitochondrial oxidative activity, resulting in adverse effects on energy expenditure, fat storage, and weight gain (70).

Thirdly, PM_2.5_ can penetrate the central nervous system by means of neurons in olfactory epithelium cells. This can disturb the control of hunger and energy expenditure by triggering inflammatory responses in the hypothalamus and causing resistance to leptin, ultimately resulting in obesity. An animal study has shown that short-term exposure to PM_2.5_ can significantly enhance inflammation markers such as Toll-like receptor 4 (Tlr4) and NF-κB kinase (Ikbke) in the hypothalamus of mice, leading to metabolic consequences similar to those induced by a high-fat diet (71). Long-term exposure to PM_2.5_ can lead to leptin resistance, insufficient levels of satiety markers, increased appetite, reduced energy expenditure, energy imbalance, and increased fat accumulation, resulting in obesity (71).

Ultimately, an imbalance in the gut microbiota could potentially act as a mediator in the relationship between PM_2.5_ and metabolic disorders. Animal and human studies provide evidence that the ingestion of air pollutants can result in changes in the diversity and relative abundance of gut microbiota, thereby reducing intestinal barrier integrity and increasing gastrointestinal inflammation (72). Multiple investigations have observed specific gut microbiota profiles in individuals with obesity and glucose and lipid metabolism disorders, suggesting that gut microbiota is involved in many physiological processes related to metabolism (73). Moreover, population-based epidemiological research evidence also confirms that the gut microbiota partially mediates the impact of PM_2.5_ on obesity and glucose and lipid metabolism disorders (74, 75).

As far as we know, this is the initial instance where an MR Framework has been employed to evaluate the genetic causality between PM_2.5_ concentrations and obesity, as well as related markers of glycolipid metabolism. This approach complements traditional observational studies and provides further evidence to assess the health hazards tied to environmental pollutants. This MR study possesses several remarkable benefits. Initially, we eliminated genetic variations commonly detected in epidemiological research that other causes could potentially influence and specifically chose those that are closely correlated with PM_2.5_-related SNPs. Furthermore, the substantial sample size utilized in our MR analysis significantly enhanced our statistical power and yielded robust evidence supporting the presence of correlations. Besides, we performed comprehensive sensitivity analyses to validate the dependability of these findings. All of the F-statistics exceed 10, suggesting a minimal likelihood of weak instrumental bias. In addition, the FDR correction method was employed to adjust for multiple tests, reducing the likelihood of Type I errors. This approach enables us to balance identifying genuine correlations and managing false positives. Given the need to compare seven groups in this investigation, the use of the Bonferroni correction is considered excessively cautious and stringent. While the Bonferroni method minimizes the incidence of Type I errors, it also decreases the power to detect genuine associations when handling a high number of comparisons, leading to an increased occurrence of false negatives. Ultimately, we employed MVMR to examine the immediate influence of PM_2.5_ on obesity and its associated indices after controlling for external smoke exposure, physical activity, EA, participation in sports clubs or gym leisure activities, and TDI.

Although there are advantages, there remain limitations. First, due to reliance on summary-level data from the GWAS database, evaluating the non-linear correlation between PM_2.5_ and obesity and related indicators was impossible. Secondly, although we have conducted multiple sensitivity analyses to detect potential pleiotropy rigorously, it must be acknowledged that no method can completely eliminate the possibility of pleiotropy, which is a limitation of MR analysis methods. Therefore, considering the potential for residual pleiotropy effects, we emphasize that our findings should be interpreted with caution. It is necessary to use additional datasets for further validation research in the future. Thirdly, our study used the IVW method as the primary statistical approach. When there is no heterogeneity and pleiotropy, the IVW method outperforms the MR Egger and weighted median methods, providing reliable conclusions. However, the differences in results between the IVW analysis and other alternative methods still deserve careful consideration. It is necessary to use larger datasets for further research in the future to verify these findings. Finally, it is worth noting that the participants in this study were all of European ancestry. While this choice minimizes potential stratification bias, it also limits the generalizability of our results to other racial groups. Due to the lack of available PM_2.5_ GWAS data from other ethnicities, we were unable to explore the association between PM_2.5_ and obesity, as well as related indicators, in other ethnic groups. We anticipate future updates of GWAS data to comprehensively investigate the impact of PM_2.5_ on obesity and metabolic disorders across diverse populations.

## 5 Conclusion

The conclusions of our MR study strongly support the existence of a significant positive association between genetically predicted PM_2.5_ concentration and pancreas fat, HbA1c, and FGF21 levels. However, the specific processes behind this link require more exploration. The results from the UVMR and MVMR analyses present limited evidence of causal links between the presence of PM_2.5_ and obesity, VAT, ASAT, or TG. This points out that other factors might have swayed past observational studies and need to be confirmed by additional investigations. Our research findings have the potential to impact public health by enhancing people’s understanding of the correlation between air quality and obesity, along with accompanying metabolic disorders. These discoveries have substantial ramifications for obesity prevention.

## Data availability statement

The original contributions presented in the study are included in the article/Supplementary material, further inquiries can be directed to the corresponding author.

## Author contributions

TW: Conceptualization, Writing – original draft. XH: Formal analysis, Methodology, Writing – review & editing. CL: Methodology, Supervision, Writing – review & editing. NZ: Funding acquisition, Visualization, Writing – review & editing. JM: Funding acquisition, Supervision, Writing – review & editing.

## Footnotes

^1^https://www.who.int/news-room/fact-sheets/detail/obesity-and-overweight, accessed on 2 January 2024.

^2^http://www.phenoscanner.medschl.cam.ac.uk/
