# Supplementary material for: A causal relationship between particulate matter 2.5 and obesity and its related indicators: a Mendelian randomization study of European ancestry
Source: Front Public Health. 2024 Jun 14;12:1366838. doi: 10.3389/fpubh.2024.1366838 (PMC11211571; doi:10.3389/fpubh.2024.1366838)
Supplement: Supplementary file 1 [file Data_Sheet_1.ZIP › supplementary material/Supplementary Figures S1-7.docx]

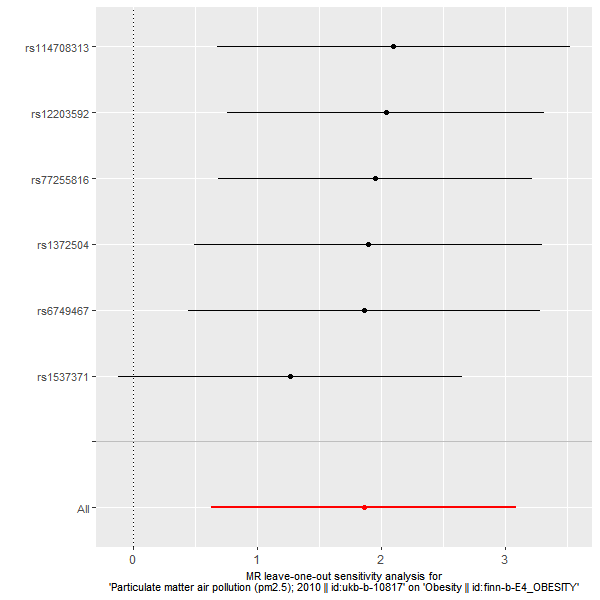
Figure S1 Leave-one-out sensitivity analysis of the causal effect of PM_2.5_ and Obesity.


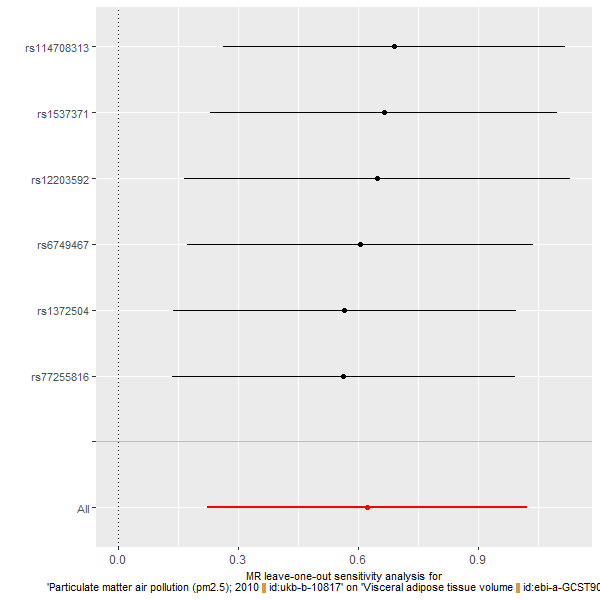
Figure S2 Leave-one-out sensitivity analysis of the causal effect of PM_2.5_ and VAT.


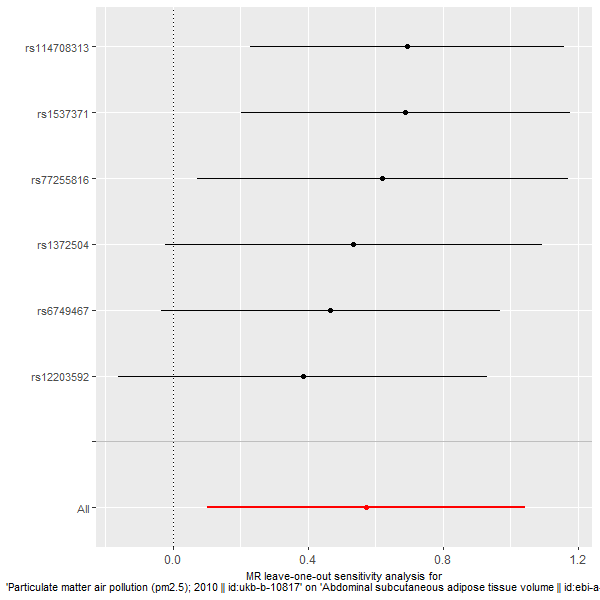
Figure S3 Leave-one-out sensitivity analysis of the causal effect of PM_2.5_ and ASAT.


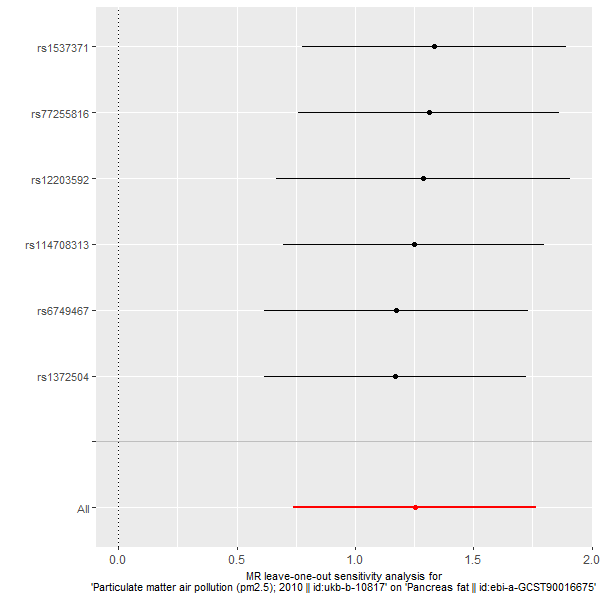


Figure S4 Leave-one-out sensitivity analysis of the causal effect of PM_2.5_ and Pancreatic fat.


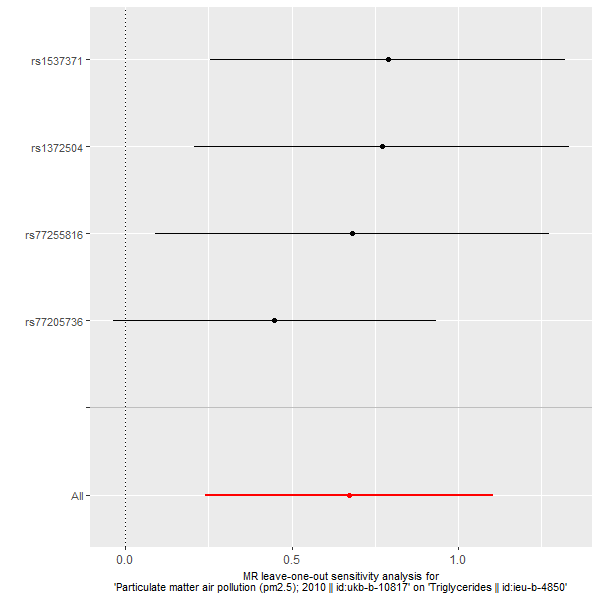
Figure S5 Leave-one-out sensitivity analysis of the causal effect of PM_2.5_ and TG.


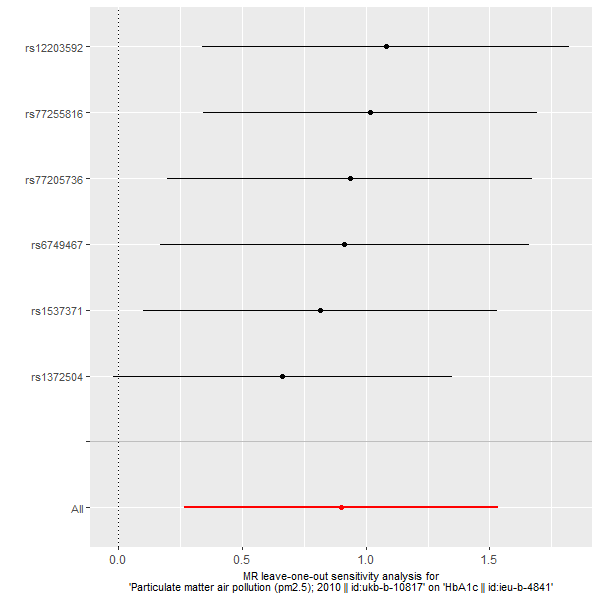
Figure S6 Leave-one-out sensitivity analysis of the causal effect of PM_2.5_ and HbA1c.


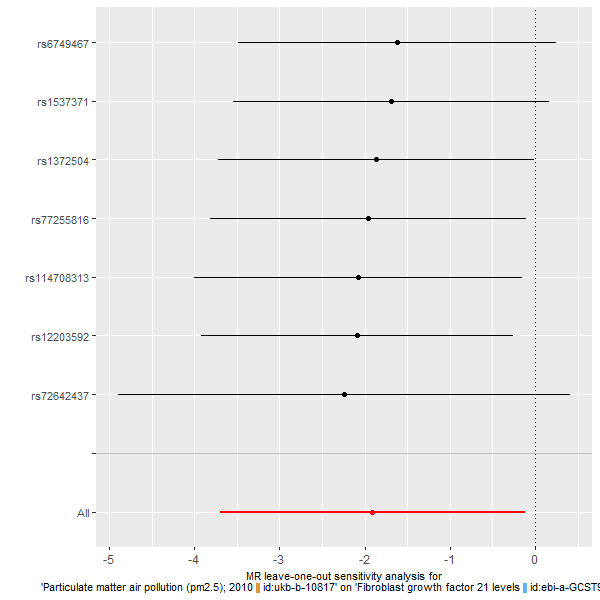
Figure S7 Leave-one-out sensitivity analysis of the causal effect of PM_2.5_ and FGF21.
