# Supplementary material for: A causal relationship between particulate matter 2.5 and obesity and its related indicators: a Mendelian randomization study of European ancestry
Source: Front Public Health. 2024 Jun 14;12:1366838. doi: 10.3389/fpubh.2024.1366838 (PMC11211571; doi:10.3389/fpubh.2024.1366838)
Supplement: Supplementary file 1 [file Data_Sheet_1.ZIP › supplementary material/Tables and figures.docx]

**Table 1. Details of studies included in Mendelian randomization (MR) analyses.**

| Traits | Author | GWAS ID | Sample size（cases/controls） | Number of SNPs | Sex | Ancestry | Year | PMID |
| --- | --- | --- | --- | --- | --- | --- | --- | --- |
| Exposure |  |  |  |  |  |  |  |  |
| PM2.5 | Ben Elsworth | ukb-b-10817 | 423,796 | 9,851,867 | Males and Females | European | 2018 | 27089921 |
| Exposure to tobacco smoke outside home | Neale | ukb-a-20 | 286,550 | 10,894,596 | Males and Females | European | 2017 | NA |
| physical activity | Neale | ukb-a-485 | 335,599 | 10,894,596 | Males and Females | European | 2017 | NA |
| Year ended full time education | Ben Elsworth | ukb-b-2709 | 112,569 | 9,851,867 | Males and Females | European | 2018 | NA |
| Leisure/social activities: Sports club or gym | Ben Elsworth | ukb-b-4000 | 461,369 | 9,851,867 | Males and Females | European | 2018 | NA |
| TDI | Neale | ukb-a-44 | 336,798 | 10,894,596 | Males and Females | European | 2017 | NA |
| Outcomes |  |  |  |  |  |  |  |  |
| Obesity | NA | finn-b-E4_OBESITY | 8,908/209,827 | 16,380,465 | Males and Females | European | 2021 | NA |
| VAT | Liu Y | ebi-a-GCST90016671 | 32,860 | 9,275,407 | NA | European | 2021 | 34128465 |
| ASAT | Liu Y | ebi-a-GCST90016672 | 32,860 | 9,275,407 | NA | European | 2021 | 34128465 |
| Pancreas fat | Liu Y | ebi-a-GCST90016675 | 25,617 | 9,275,407 | NA | European | 2021 | 34128465 |
| Triglycerides | Howe LJ | ieu-b-4850 | 78,700 | 7,892,037 | Males and Females | European | 2022 | NA |
| HbA1c | Howe LJ | ieu-b-4841 | 17,724 | NA | Males and Females | European | 2022 | NA |
| FGF21 | Gilly A | ebi-a-GCST90010123 | 1,298 | 18,160,173 | NA | European | 2020 | 33303764 |

PM2.5, particulate matter 2.5; TDI, Townsend deprivation index at recruitment; VAT, visceral adipose tissue volume;ASAT, abdominal subcutaneous adipose tissue volume; HbA1c , glycosylated hemoglobin ; FGF21, fibroblast growth factor 21 levels.

**Table 2.** **Heterogeneity, horizontal pleiotropy, and MR-PRESSO tests of the associations between PM_2.5_ and obesity and its related indicators.**

| Outcomes | Pleiotropy test | | |  | Heterogeneity test | | | | | | |  | MR-PRESSO |
| --- | --- | --- | --- | --- | --- | --- | --- | --- | --- | --- | --- | --- | --- |
|  | MR-Egger | | |  | MR-Egger | | |  | Inverse-variance weighted | | |  | Global Test |
|  | Intercept | SE | *P* |  | Q-value | Q-df | Q-pval |  | Q-value | Q-df | Q-pval |  | *P*value |
| Obesity | 0.044 | 0.032 | 0.239 |  | 2.294 | 4 | 0.682 |  | 4.204 | 5 | 0.520 |  | 0.563 |
| VAT | 0.001 | 0.010 | 0.905 |  | 1.799 | 4 | 0.773 |  | 1.816 | 5 | 0.874 |  | 0.899 |
| ASAT | 0.005 | 0.013 | 0.727 |  | 5.689 | 4 | 0.224 |  | 5.888 | 5 | 0.317 |  | 0.349 |
| Pancreas fat | 0.007 | 0.013 | 0.581 |  | 1.423 | 4 | 0.840 |  | 1.783 | 5 | 0.878 |  | 0.908 |
| Triglycerides | -0.002 | 0.014 | 0.885 |  | 3.236 | 2 | 0.198 |  | 3.280 | 3 | 0.350 |  | 0.428 |
| HbA1c | 0.018 | 0.011 | 0.198 |  | 1.913 | 4 | 0.752 |  | 4.627 | 5 | 0.463 |  | 0.483 |
| FGF21 | -0.030 | 0.032 | 0.385 |  | 2.037 | 5 | 0.844 |  | 2.942 | 6 | 0.816 |  | 0.870 |

PM2.5, particulate matter 2.5; VAT, visceral adipose tissue volume;ASAT, abdominal subcutaneous adipose tissue volume; HbA1c, glycosylated hemoglobin; FGF21, fibroblast growth factor 21 levels; MR-PRESSO, Mendelian randomization pleiotropy residual sum and outlier; Q-value, the statistics of Cochran's Q test; SE, standard error.

**Table 3.** **Causal estimates of PM_2.5_ on obesity and its related indicators in MVMR-IVW.**

| Outcome | MVMR | Method | *P*value | OR | Low | UP |
| --- | --- | --- | --- | --- | --- | --- |
| Obesity | IVW | Adjusted for Exposure to tobacco smoke | 0.598 | 1.254 | 0.541 | 2.907 |
|  |  | Adjusted for Strenuous sports in last 4 weeks | 0.498 | 1.303 | 0.606 | 2.800 |
|  |  | Adjusted for Year ended full time education | 0.454 | 1.368 | 0.602 | 3.106 |
|  |  | Adjusted for Sports club or gym activities | 0.606 | 1.231 | 0.559 | 2.710 |
|  |  | Adjusted for Townsend deprivation index | 0.886 | 0.931 | 0.349 | 2.481 |
|  |  | Adjusted for all | 0.401 | 1.642 | 0.516 | 5.228 |
| VAT | IVW | Adjusted for Exposure to tobacco smoke | 2.44E-06* | 1.799 | 1.409 | 2.297 |
|  |  | Adjusted for Strenuous sports in last 4 weeks | 0.011* | 1.737 | 1.137 | 2.651 |
|  |  | Adjusted for Year ended full time education | 4.03E-12* | 2.028 | 1.661 | 2.476 |
|  |  | Adjusted for Sports club or gym activities | 0.018* | 1.621 | 1.085 | 2.421 |
|  |  | Adjusted for Townsend deprivation index | 0.326 | 1.263 | 0.793 | 2.012 |
|  |  | Adjusted for all | 0.233 | 1.373 | 0.816 | 2.311 |
| ATST | IVW | Adjusted for Exposure to tobacco smoke | 0.203 | 1.408 | 0.832 | 2.382 |
|  |  | Adjusted for Strenuous sports in last 4 weeks | 0.454 | 1.289 | 0.664 | 2.501 |
|  |  | Adjusted for Year ended full time education | 0.001* | 1.840 | 1.268 | 2.670 |
|  |  | Adjusted for Sports club or gym activities | 0.059 | 1.555 | 0.984 | 2.458 |
|  |  | Adjusted for Townsend deprivation index | 0.887 | 0.963 | 0.571 | 1.622 |
|  |  | Adjusted for all | 0.700 | 1.130 | 0.608 | 2.101 |
| Pancreas fat | IVW | Adjusted for Exposure to tobacco smoke | 5.42E-09* | 3.116 | 2.127 | 4.565 |
|  |  | Adjusted for Strenuous sports in last 4 weeks | 3.40E-10* | 3.382 | 2.312 | 4.947 |
|  |  | Adjusted for Year ended full time education | 5.43E-18* | 3.347 | 2.545 | 4.402 |
|  |  | Adjusted for Sports club or gym activities | 2.88E-06* | 3.029 | 1.904 | 4.817 |
|  |  | Adjusted for Townsend deprivation index | 7.10E-04* | 3.388 | 1.672 | 6.868 |
|  |  | Adjusted for all | 9.82E-05* | 3.612 | 1.893 | 6.892 |
| TG | IVW | Adjusted for Exposure to tobacco smoke | 0.038* | 1.665 | 1.030 | 2.691 |
|  |  | Adjusted for Strenuous sports in last 4 weeks | 0.010* | 1.720 | 1.136 | 2.640 |
|  |  | Adjusted for Year ended full time education | 0.878 | 1.047 | 0.580 | 1.891 |
|  |  | Adjusted for Sports club or gym activities | 3.13E-04* | 2.775 | 1.593 | 4.835 |
|  |  | Adjusted for Townsend deprivation index | 0.180 | 1.541 | 0.819 | 2.898 |
|  |  | Adjusted for all | 0.597 | 1.306 | 0.486 | 3.514 |
| HbA1c | IVW | Adjusted for Exposure to tobacco smoke | 0.002* | 3.095 | 1.494 | 6.411 |
|  |  | Adjusted for Strenuous sports in last 4 weeks | 0.003* | 3.154 | 1.464 | 6.794 |
|  |  | Adjusted for Year ended full time education | 2.00E-04* | 3.058 | 1.686 | 5.545 |
|  |  | Adjusted for Sports club or gym activities | 3.00E-04* | 2.775 | 1.593 | 4.835 |
|  |  | Adjusted for Townsend deprivation index | 1.90E-04* | 5.240 | 2.197 | 12.497 |
|  |  | Adjusted for all | 9.00E-05* | 5.429 | 2.327 | 12.665 |
| FGF21 | IVW | Adjusted for Exposure to tobacco smoke | 0.003* | 0.108 | 0.025 | 0.469 |
|  |  | Adjusted for Strenuous sports in last 4 weeks | 0.010* | 0.138 | 0.030 | 0.628 |
|  |  | Adjusted for Year ended full time education | 0.053 | 0.230 | 0.052 | 1.018 |
|  |  | Adjusted for Sports club or gym activities | 0.001* | 0.128 | 0.036 | 0.453 |
|  |  | Adjusted for Townsend deprivation index | 0.023* | 0.162 | 0.034 | 0.776 |

PM2.5, particulate matter 2.5; VAT, visceral adipose tissue volume;ASAT, abdominal subcutaneous adipose tissue volume; HbA1c, glycosylated hemoglobin; FGF21, fibroblast growth factor 21 levels; MVMR, Multivariable Mendelian randomization; SE, standard error; OR, odds ratio; CI, confidence interval; *indicates that the relationship has statistical significance.

**Figure 1 Assumption of the Mendelian randomization (MR) analysis for PM_2.5_ and obesity and its related indicators.**

**
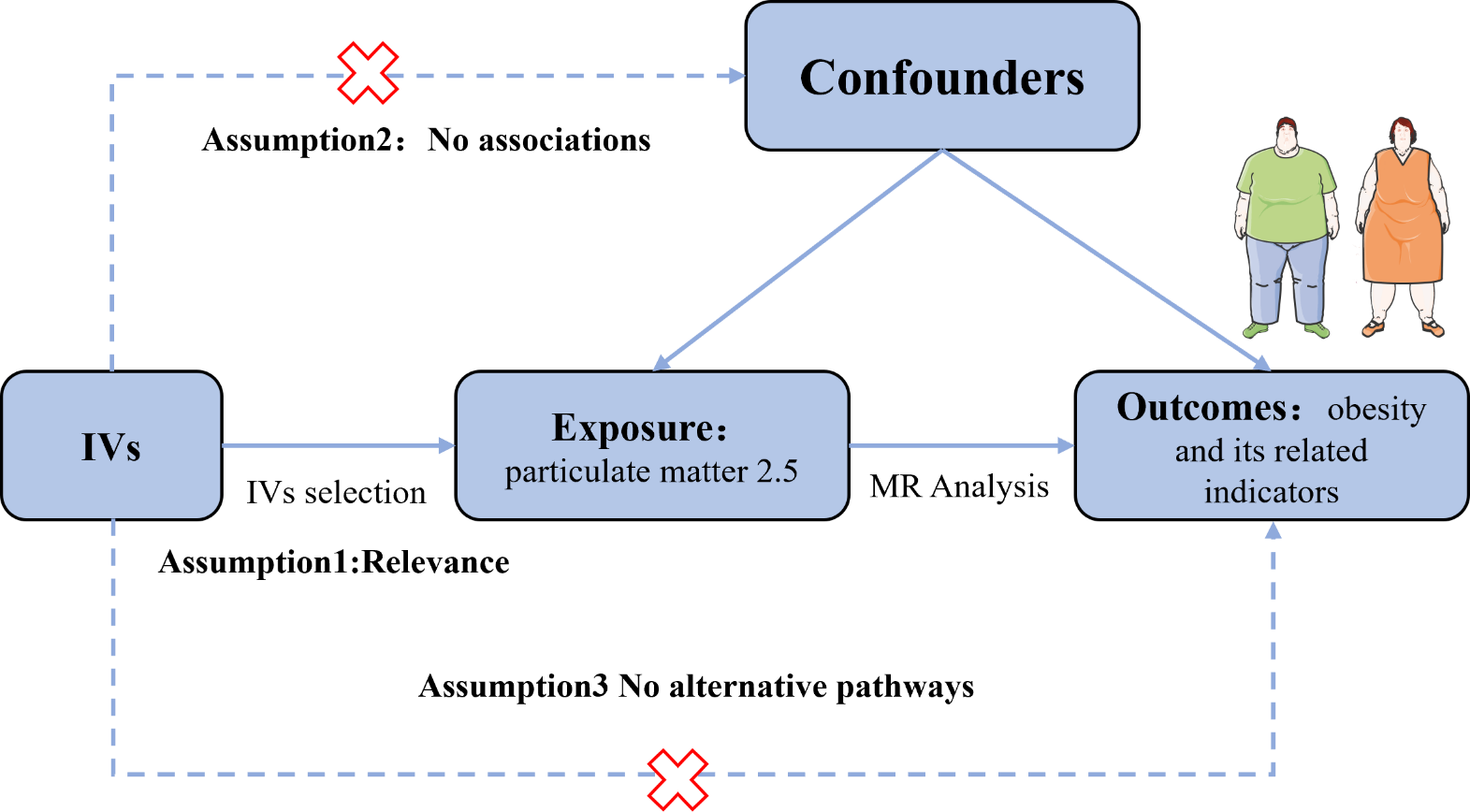
**

**Figure 2 Association of genetically predicted PM2.5 and obesity and its related indicators.**

**
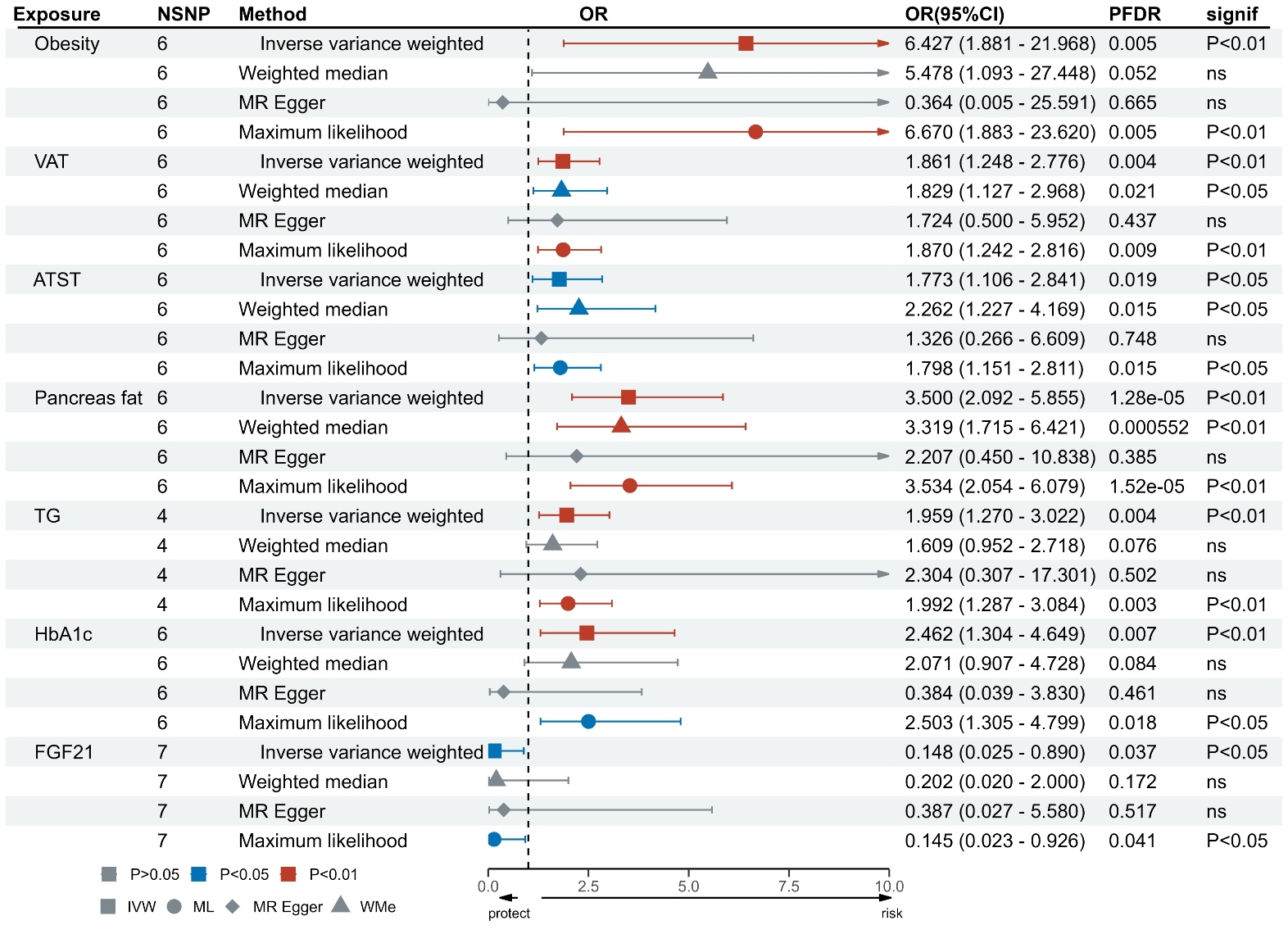
**

**
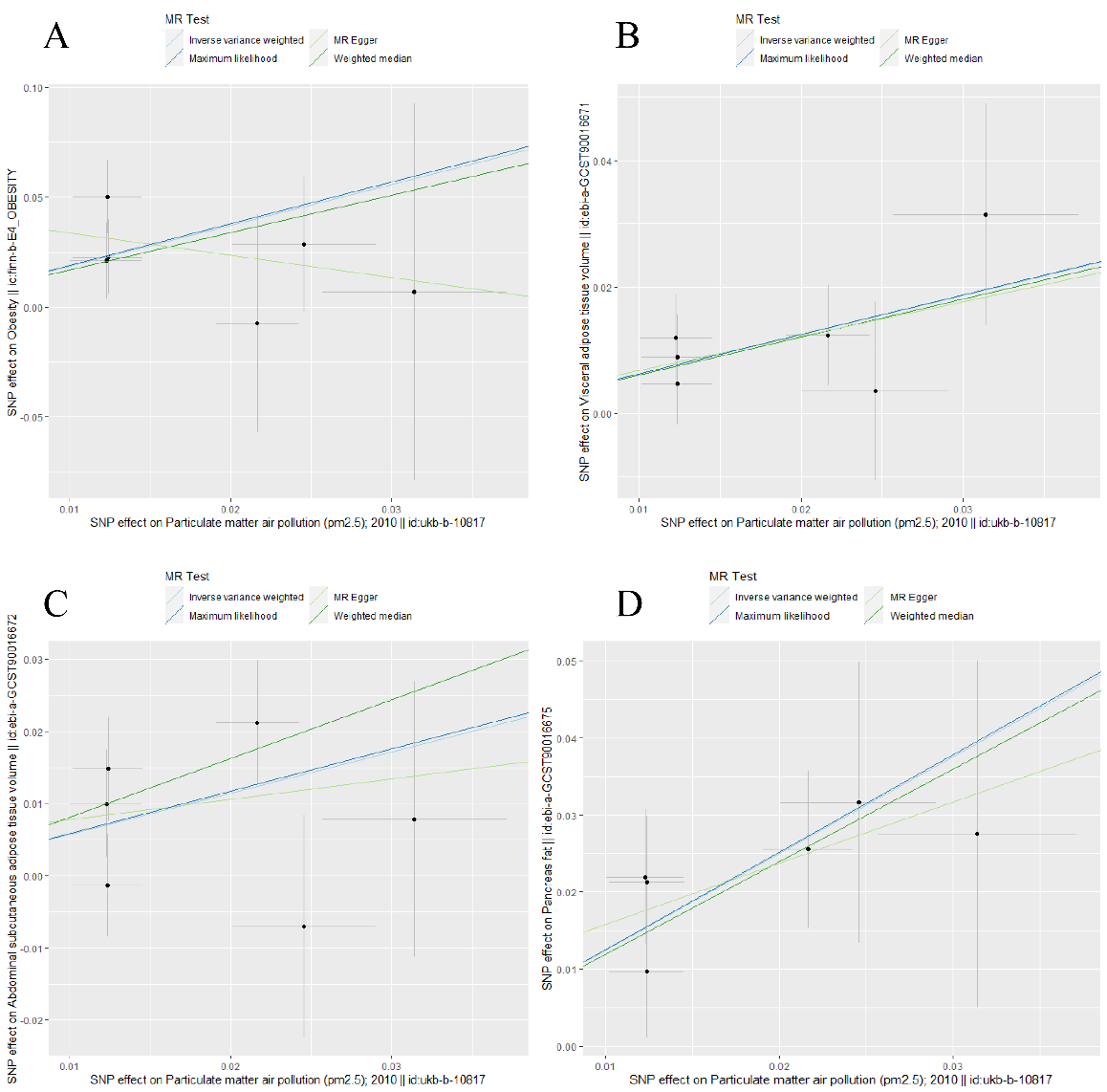
Figure 3 Scatter plots for Mendelian randomization (MR) analyses of the correlation between PM_2.5_ and obesity and its related indicators. (A) obesity; (B)VAT; (C) ASAT; (D) Pancreatic fat.**

**Figure 4 Scatter plots for Mendelian randomization (MR) analyses of the correlation between PM_2.5_ and obesity and its related indicators. (A) TG; (B) HbA1c; (C) FGF21.**

**
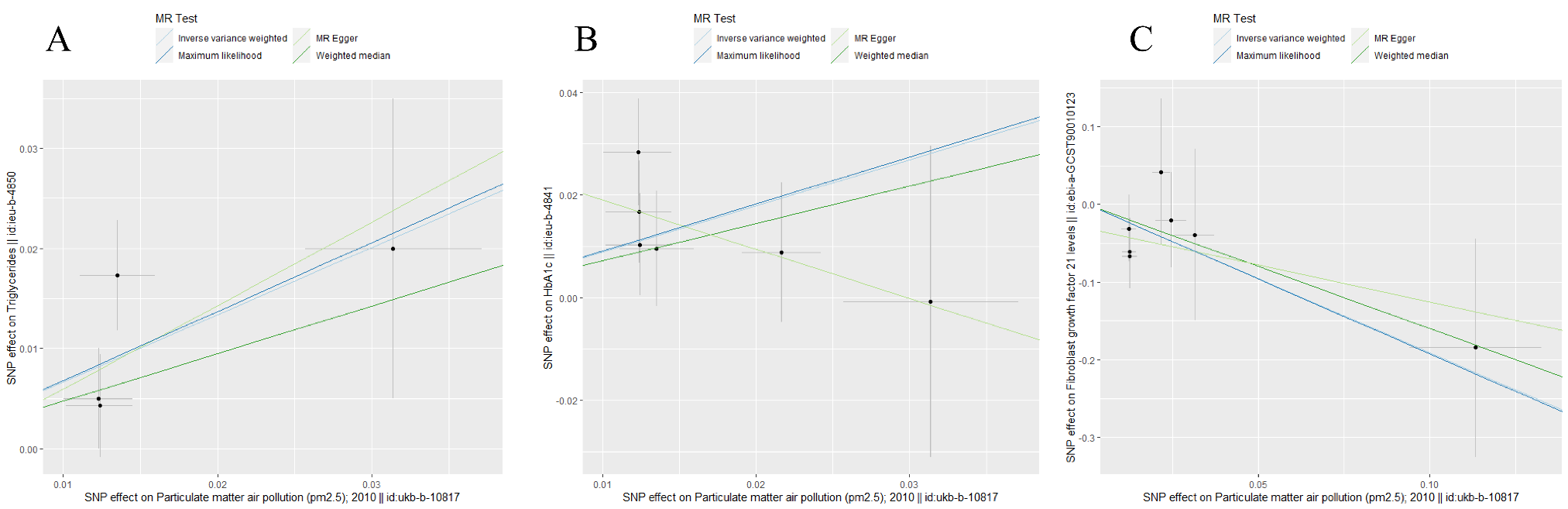
**
