# Supplementary figures and images for: A causal relationship between particulate matter 2.5 and obesity and its related indicators: a Mendelian randomization study of European ancestry
Source: Front Public Health. 2024 Jun 14;12:1366838. doi: 10.3389/fpubh.2024.1366838 (PMC11211571; doi:10.3389/fpubh.2024.1366838)

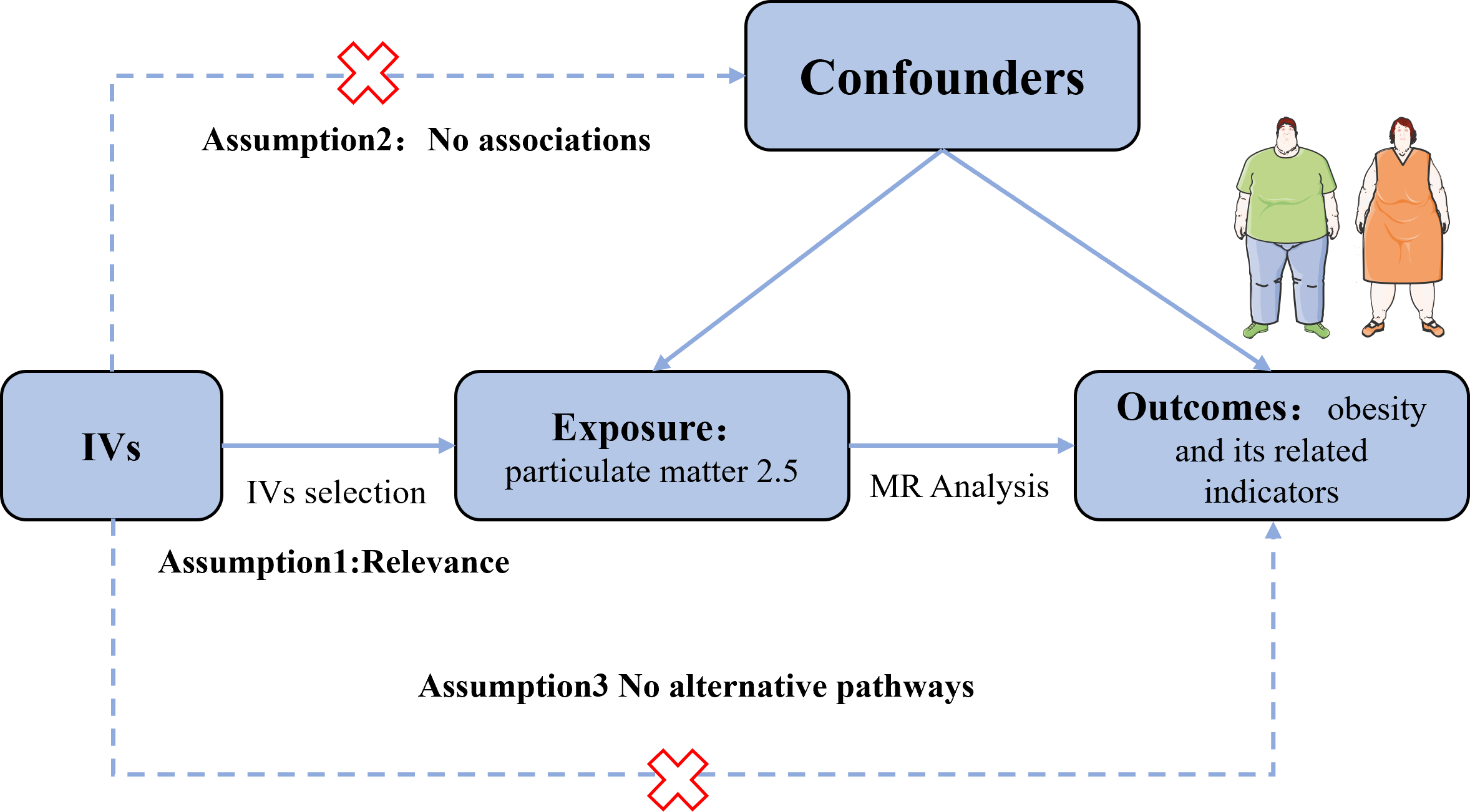

Supplement: Supplementary file 1 [file Data_Sheet_1.ZIP › supplementary material/Figure1.tif]

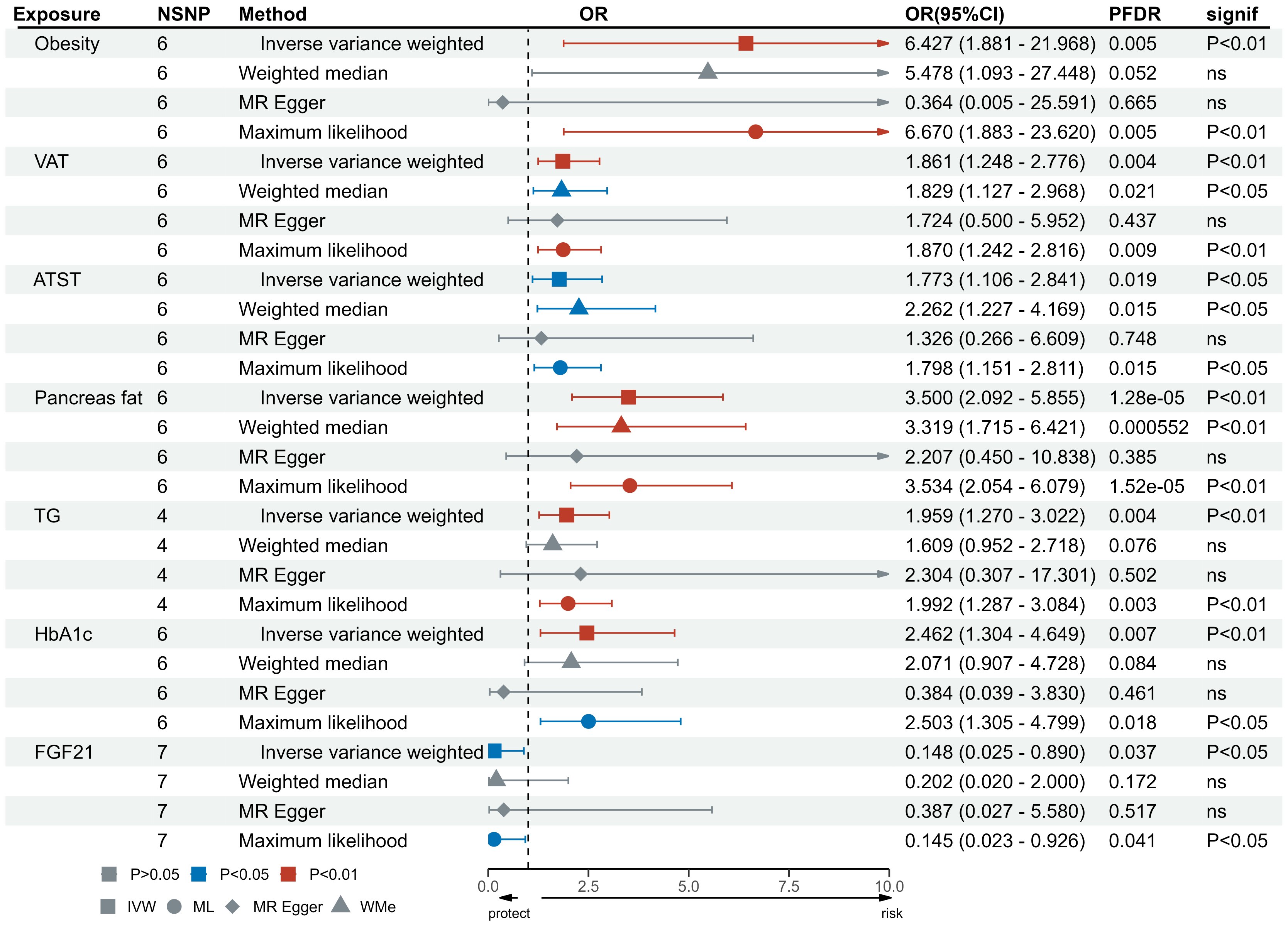

Supplement: Supplementary file 1 [file Data_Sheet_1.ZIP › supplementary material/Figure2.tif]
